# Exploring patient and health care provider perspectives on barriers to diabetic retinopathy screening in public health facilities in North India

**DOI:** 10.1038/s41598-025-92795-y

**Published:** 2025-03-10

**Authors:** Anshul Chauhan, Mona Duggal, Ankita Kankaria, Vishali Gupta, Shrutika Dhiman, Mandeep Singh, Basavaraj Tigari, Sanjay Bhadada, Luke Vale

**Affiliations:** 1 Advanced Eye Centre (AEC), Post Graduate Institute of Medical Education and Research, Madhya Marg, Sector- 12, Chandigarh, 160012 India; 2 Department of Community Medicine and Family Medicine, All India Institute of Medical Sciences, Bathinda, Punjab India; 3 Global Health Economics Centre, London School of Hygiene and Tropical Medicine, Tavistock Place, London, UK

**Keywords:** Barriers, Diabetic retinopathy screening, Public health facilities, North India, Endocrinology, Health care, Medical research, Risk factors

## Abstract

Diabetic retinopathy (DR), a prevalent microvascular complication of diabetes mellitus (DM), can be prevented with early detection and timely intervention. DR is asymptomatic in its early stages, highlighting the importance of screening for accurate referral and effective management. Multiple barriers impede access to diabetic retinopathy screening (DRS), creating significant public health challenges in regions with high DM prevalence. This study explores the perspectives of people with DM (PwDM) and healthcare providers (HCP) on these barriers. A qualitative study using in-depth interviews (IDI) was conducted between October 2022 and January 2023 in Punjab and Chandigarh. Through purposive sampling, IDIs were conducted with 7 PwDM and 19 HCPs, including retina specialists, ophthalmologists, optometrists, medical officers (MO), Community Health Officers (CHO), and ASHA workers from various public health facilities. A semi-structured topic guide facilitated the interviews, and thematic analysis was applied, utilizing the healthcare access barrier (HCAB) model as a framework. The study identified financial barriers due to insurance unawareness and employment constraints. Structural challenges included insufficient DRS infrastructure, untrained staff, the need for accompaniment, and limited access to screening sites. Limited awareness and misconceptions about DR characterized cognitive barriers, while psychological barriers involved mistrust of the health system, anxiety, and frustration from low vision. Addressing these issues is essential to improve DRS uptake and eye health outcomes. Managing diabetes and VTDR is challenging, highlighting the need for community-level DRS. Enhancing DR awareness and promoting public health insurance benefits are crucial for overcoming barriers and improving screening rates.

**Supplementary Information:**

The online version contains supplementary material available at 10.1038/s41598-025-92795-y.

## Introduction

Diabetes mellitus (DM) is a growing global epidemic, projected to affect 783 million people by 2045^1^. Diabetic retinopathy (DR) is a severe microvascular complication of DM that significantly contributes to vision loss and irreversible blindness^2^. A systematic review by Flaxman et al. (2017) highlighted an increase in blindness due to DR from 1990 to 2015^3^. DR has globally emerged as a significant public health challenge, with vision-threatening diabetic retinopathy (VTDR) affecting 5% of people with diabetes mellitus (PwDM), which is anticipated to increase with the growing diabetic population^4^.

Unlike conditions such as cataracts, refractive errors, or corneal blindness, patients with DR may maintain normal visual acuity despite significant changes occurring in the retina.

This means that by the time symptoms appear, extensive changes from DR have already occurred^5^. Timely screening for early detection and effective treatment can prevent approximately 50–70% of visual impairments associated with DR^6^.

In India, managing DR is challenged by inadequate screening programs, low public awareness, and limited understanding of the need for regular retinal exams^7^. Most retinal services are privately funded, with significant variation in healthcare quality^8^. A study on PwDM in India by Shukla et al. (2016) showed that 45% experienced vision loss before their initial visit to an eye facility and before a DR diagnosis^8^. A screening study (by Lingam et al. (2018) found that 2% of PwDM at the tertiary level, 40% at the secondary level, and 50% at the primary level had never undergone a previous dilated eye examination^9^.

Despite ongoing efforts to enhance routine diabetic retinopathy screening (DRS) and follow-up for better vision outcomes, screening and follow-up uptake remain inadequate^10–14^. Studies on eye health-seeking behaviour and barriers to DRS for PwDM have identified context-specific sociocultural factors, including low economic status^15,16^, low literacy, lack of screening awareness^17^, and socioeconomic disparities^18^. Barriers include cultural beliefs, denial, fear, hopelessness^18^, perception of healthy eyes, and lack of companions to access healthcare facilities^14^. Additional barriers to accessing DRS in India included old age, physical disability, long distances to healthcare centres, and travel expenses^8^, and having regional variations^19^.

Few qualitative studies have examined barriers to accessing DRS, particularly within India’s public health settings. This gap highlights the importance of our research, which investigated the perspectives of PwDM and healthcare providers (HCPs) on these barriers. Our study employs the Health Care Access Barriers (HCAB)^20^ model to explore perceptions related to access to DRS services.

### Conceptual framework

The HCAB model provides a framework for identifying, categorizing, and addressing healthcare access barriers by focusing on three modifiable financial, structural, and cognitive categories to enhance accessibility to healthcare services. (Supplementary Fig. 1a and 1b) Financial barriers relate to the cost of care and health insurance status; structural barriers describe challenges within institutions and organizations; and cognitive barriers are limitations in knowledge and communication. The model focuses on identifying measurable and modifiable healthcare access barriers and intermediary factors that connect these barriers to health outcomes. It highlights how access barriers contribute to reduced screening rates, delayed care, and inadequate treatment, leading to poor health outcomes and exacerbating health disparities^20–22^.Using healthcare access barriers as units of analysis, the model explores the causal pathways linking these barriers to adverse health outcomes^20^. Psychological barriers were incorporated to include the emotional dimension alongside the three primary themes^23^.

## Methods

This manuscript has been prepared in accordance with the Consolidated Criteria for Reporting Qualitative Research (COREQ) guidelines, which ensure comprehensive and transparent qualitative study reporting^24^ (see checklist Supplementary Table 1).

### Study participants and sampling

The study was conducted from October 2022 to January 2023. The study included two distinct categories of participants: the PwDM and the HCP. The PwDM and the HCP were purposely sampled from different public health facilities, including primary (community), secondary (district hospitals (DH)), and tertiary healthcare settings (retina unit). Maximum variation sampling was employed to recruit participants from both groups with diverse experiences accessing and providing DRS screening services at different levels of public health facilities. The PwDM, for five years or more duration of diabetes, were identified with the help of Accredited Social Health Activist (ASHA) workers in the community, ophthalmologists in DH, and retina specialists at a retina unit of a tertiary healthcare centre. Of 28 approached participants, 26 consented, while two PwDM declined, citing time constraints. The DH here is referred to as the civil hospital Mohali, Punjab, and the tertiary centre is the Post Graduate Institute of Medical Education and Research (PGIMER), Chandigarh, India.

The second group of participants consisted of HCPs involved in delivering DM and eye care services at various levels of public health settings in Punjab and Chandigarh. Twenty HCPs from the Mohali district were purposefully selected with assistance from a Senior Medical Officer (SMO). The group included Medical Officers (MO) from three Primary Health Centres (PHCs), Community Health Officers (CHO) from five Health and Wellness Centres (HWCs), optometrists from a Community Health Centre (CHC), ophthalmologists from a District Hospital (DH), and retina specialists from a tertiary healthcare retina unit. Accredited Social Health Activists (ASHAs) were recruited with the help of support from the PHC Medical Officer (MO). Three HCPs declined participation due to time constraints, and two additional ophthalmologists from the Moga and Faridkot districts of Punjab were included upon recommendation.

### Ethics and inclusion criteria

The study received approval from the Postgraduate Institute of Medical Education and Research (PGIMER) Institutional Ethics Committee (PGI/IEC/2020/000741) (PGI/IEC/2020/000741)and followed the recommendations of the Declaration of Helsinki. The study was prospectively registered with the Clinical Trials Registry India (CTRI/2022/10/046283).

### Design

This qualitative study used a cross-sectional study design to explore the barriers to accessing DRS services in public health facilities in northern India. Semi-structured interviews were conducted to gather participants perspectives on eye care-seeking behaviors, care pathways, and obstacles to DRS among people with PwDM. The in-depth interview guides were developed based on the themes of the HCAB model^20^. These guides explored participants’ financial, structural, cognitive, and psychological barriers to DRS access in public health settings. The in-depth interview guide was developed in English and later translated into Hindi and Punjabi. Open-ended questions and probes were pre-specified and logically organized, varying usage based on the interview flow. Pilot interviews with two PwDM, an ASHA, and a CHO led to revisions for clarity. The interview questions were amended to remove ambiguous or complicated words and were replaced with simpler terms to aid understanding by the study participants^25–27^ (Supplementary Table 2).

### Data collection

Two research fellows (AC, MS), both males, holding master’s degrees and pursuing doctoral studies with more than 5 years of experience in qualitative research, conducted the IDIs. PwDM were interviewed in quiet locations within the hospital or at home, while HCPs were interviewed face-to-face or via Zoom, depending on their availability. The authors employed a flexible approach to conducting IDIs, adapting to participants’ cues and modifying the questioning as needed while ensuring all key issues were thoroughly addressed. PwDM and ASHAs were interviewed in Hindi or Punjabi, whereas HCPs primarily used English. The study procedures were explained, and written informed consent was obtained from the participants for face-to-face interviews or verbal communication via Zoom. About 10/19 (50%) HCP interviews were conducted via Zoom due to work commitments. Each interview lasted 40–45 min and was audio recorded. The qualitative data was collected from respective participants until data saturation was achieved^28^ which occurred when further interviews provided no additional data^28^.

### Data analysis

**Fig. 1:**
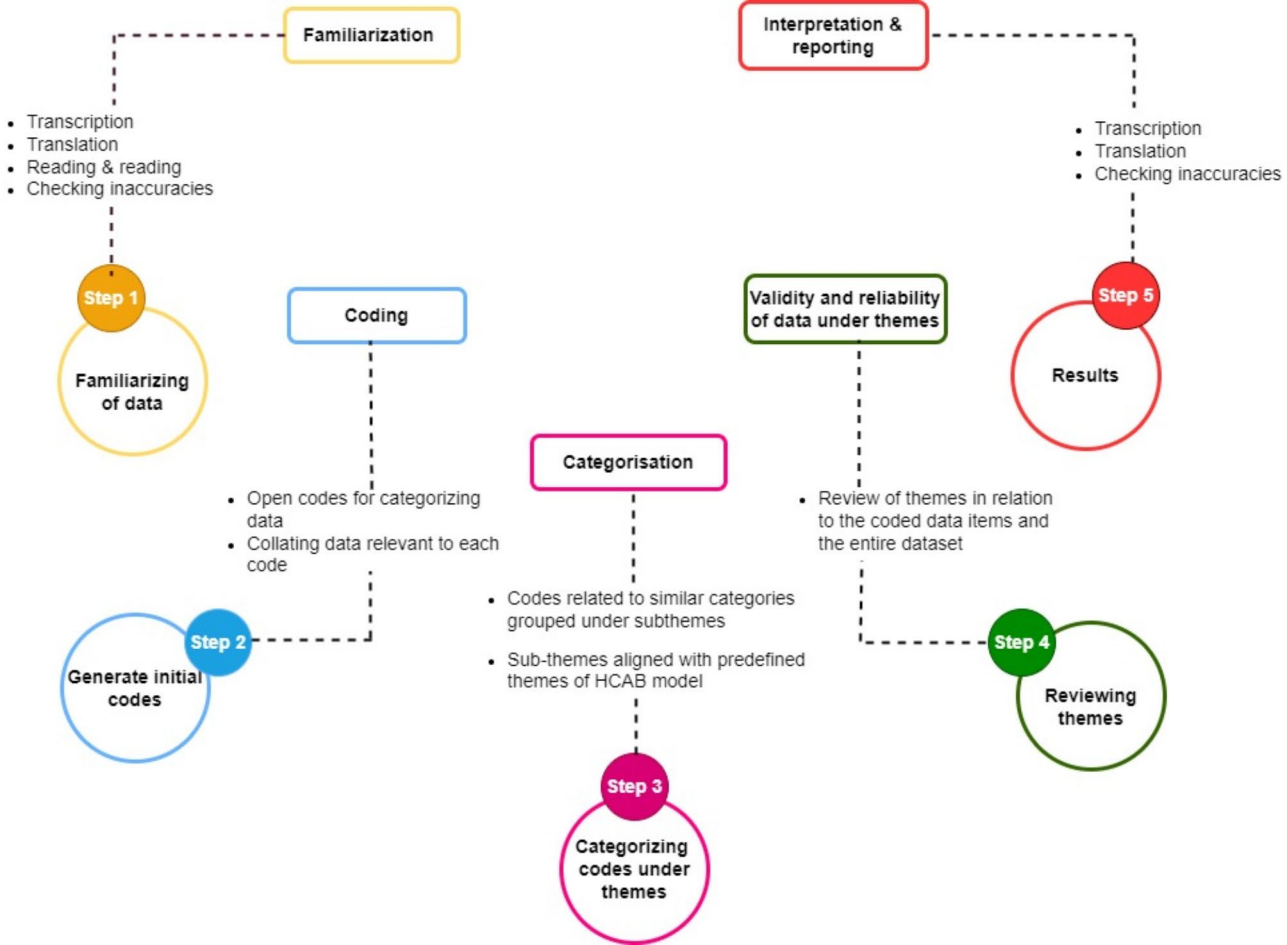
Thematic analysis steps guide by Braun and Clarke.

Two trained investigators transcribed audio-recorded interviews and translated them from Hindi and Punjabi into English. The thematic analysis was undertaken using Braun and Clarke’s six-phase approach^29^ (Fig. 1). The six phases follow a logical sequence, but the analysis was not linear; it is recursive and iterative, requiring back-and-forth forth between phases as needed^30^. Deductive thematic analysis was used to identify themes from the HCAB model, including financial, cognitive, psychological, and structural barriers^20,31^. The transcripts were also analyzed using an inductive thematic analysis to identify new codes and themes that had not been previously identified within the HCAB model^23,32^. The HCAB model was applied flexibly, allowing for adaptation to effectively present the study’s findings in the results section^20,31^. The codes were developed through an iterative process involving familiarizing with the data, reading the transcripts, data interpretation, open coding, and refining the codes through comparison among coders (AC, SD, HR). Experienced public health experts (MD, AK) merged codes with similar meanings to eliminate redundancy. Coding was performed using Atlas.Ti 23 software^32^. If any new theme emerged, it was discussed with the (MD and AK) to reach a consensus on whether it represented a new theme or aligned with one of the barriers defined by the HCAB Model. After each IDI, the research team held debriefing sessions and documented their reflections. Continuous discussions among researchers ensured that all viewpoints were incorporated into the data interpretation process.

The phases include data familiarization, initial coding, theme generation, theme review, definition and naming, and report production. In Fig. 1, steps 4 and 5 are combined and presented as Step 4: reviewing themes.

### Findings

The two groups of participants are denoted in italics with a unique ID number: P 1–26 (PwDM); Opt 1–3 (Ophthalmologists); ASHA 1–4 (ASHA workers); CHO 1–3 (Community Health Officer), Optom 1–5 (Optometrists); MO 1–2 (Medical Officers); RS 1–2 (Retina Specialists). Quotes were edited for readability to retain their original meaning; ellipses (…) show removed text.

The details on the inclusion criteria can be found in Supplementary Table 3.

### Participant characteristics

The study included 26 PwDM (M:15, F:11), and 16 PwDM were 50 or older, with an average age of 60.18 ± 11.13 years and 11.9 ± 6.3 years average duration of diabetes. Approximately 2 PwDM had a graduation degree, and 12 had no formal education. Employment status was diverse, with 5 working as daily wagers, 4 being retired government employees, and the rest unemployed (Supplementary Table 4).

The HCP consisted of 19 individuals. Of these 19, nine women and 10 men had varied professional experience and educational backgrounds. Retina specialists were ophthalmologists trained in retina sub-specialty; 2 optometrists had a master’s degree, and two ASHAs had an education up to the matriculation level. The HCP’s average age was 38.3 ± 8.86 years of professional experience (Supplementary Table 5).

### Themes of analysis

Table 1 presents an overview of the themes and subthemes derived from the data by deductive and inductive thematic analysis, per the HCAB model.

**Table 1:** The analysis of the data revealed various themes and subthemes.

Theme	Subthemes
Financial barriers	1. Affordability
Structural barriers	1. Health systems barrier 2. Individual-level barrier
Cognitive barriers	1. Perceptions about doctor-patient relationships and communications 2. Limited disease-related communication 3. Awareness and attitude about Diabetes and DR 4. Health-seeking behaviour
Psychological barriers	1 Fear and anxiety 2. Hopelessness

Representative quotes related to the HCAB model are provided in Supplementary Table 6.

**Fig. 2:**
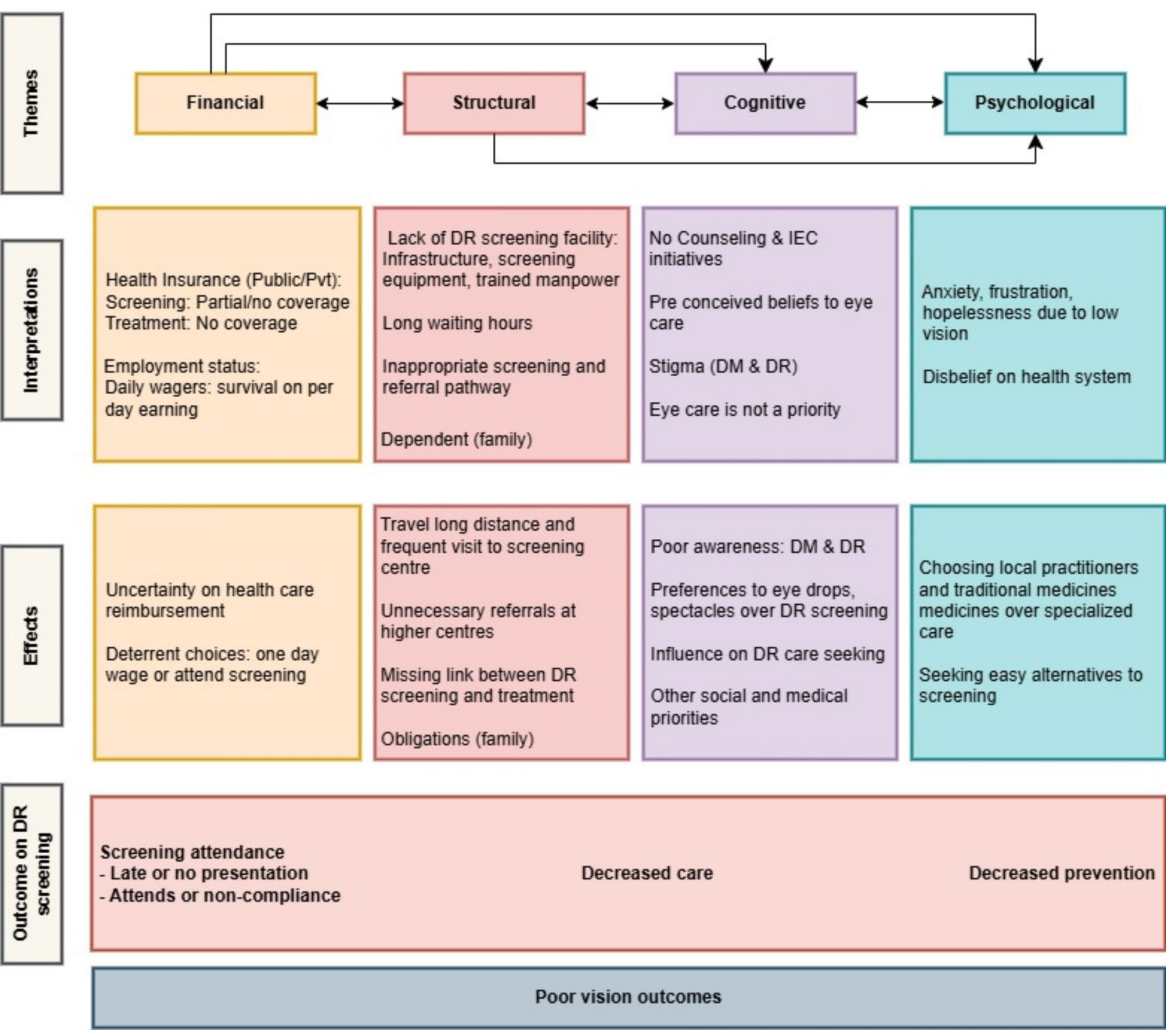
HACB model to detect diabetic retinopathy screening barriers on vision outcomes.

We found evidence of an interplay of HCAB model themes contributing to barriers to DRS (Fig. 2). The model depicts interconnected findings using directional arrows, illustrating a top tier of interwoven barriers affecting healthcare access (financial, cognitive, structural, and psychological) and a second tier showcasing interview interpretations. The third tier illustrates the impacts of tier two barriers, while the fourth tier focuses on three primary consequences (screening attendance, decreased care, and reduced prevention). The bottom level portrays the cumulative health outcomes resulting from these factors. The diagram has been modified from its original version^20,23^ this study will include interpretation of the interview and its potential effects on accessing DRS.

### PwDM and HCP perspectives

#### Theme 1: financial barriers

The cost of healthcare services is a key aspect of financial barriers in the HCAB model^20^. In our study, themes related to financial barriers included affordability, which was influenced by the PwDM’s healthcare insurance and employment status.

#### Affordability

PwDM and HCPs identified affordability (cost of treatment) as a significant barrier following DRS. This financial burden was indirectly linked to employment informality, as those in informal jobs often earned lower incomes, making healthcare costs a larger proportion of their earnings. Many individuals over 60 with DM expressed concerns about insufficient earnings to cover their eye care needs. The majority^16^ of HCPs highlighted difficulties in making decisions regarding the trade-offs between deteriorating eye health, familial obligations, and potential loss of income. Additional expenses such as boarding, lodging, travel, and food compounded the challenges for individuals accessing DRS at public health facilities. The loss of income combined with out-of-pocket medical expenses creates significant financial barriers for individuals without access to such benefits. All PwDMs were uninsured and unaware of the Pradhan Mantri Jan Arogya Yojna (PM-JAY) public health insurance coverage for DRS. Trust in private health insurance companies was minimal due to inadequate information about health packages, lack of transparency, and delayed reimbursements. HCPs also raised concerns about misleading television and social media advertisements promoting affordable DR care, discouraging PwDM from pursuing DRS but instead choosing unconventional and unscientific treatment methods.

#### Theme 2: structural barriers

Structural barriers in healthcare define service availability and accessibility. The availability of healthcare services shapes structural barriers, both within and outside healthcare facilities. These barriers include, but are not limited to, transportation, geographical location, organizational barriers, availability of services, access to health information, waiting times, and healthcare infrastructure^20,23^. The theme of Structural Barriers was divided into two sub-themes, namely, healthy system barriers and individual barriers.

#### Health systems barriers

The participants generally believed that while tertiary care centres had ample healthcare services, the availability of DRS at primary and secondary centres was limited. The PwDM and HCPs highlighted the inequitable distribution and aggregation of ophthalmologists and retina specialists only at tertiary care facilities. Some patients at the tertiary care centre expressed distress or difficulties over traveling long distances from their home state due to the lack of DRS and treatment services locally. Participants noted that the considerable distance to treatment facilities posed challenges, primarily due to the associated costs and time required for travel. Dependence on family members to access distant healthcare services contributed to disengagement from the healthcare system due to logistical difficulties, unavailability of accompanying members, and financial constraints affecting both genders. Those who accessed higher centres faced extended waiting times, with limited and brief interactions with retina specialists and ophthalmologists.

Even after reaching the facility, extended waiting hours at healthcare centres were a major barrier to timely access to ophthalmologists. PwDM reported difficulties accessing specialists due to high patient volumes, often leaving the institute without treatment. Some individuals sought assistance from optical stores for low vision caused by DR; however, this often resulted in unsatisfactory outcomes as their low vision, attributed to DR, was inappropriately addressed with spectacles. The study highlighted prolonged waiting times, particularly for the elderly, as a major source of dissatisfaction with the healthcare system and a barrier to screening services. Patients are often referred to tertiary centres due to inadequate infrastructure and specialist availability at primary and secondary facilities. This centralization of expertise necessitates patients to seek care at higher-level centres, leading to increased patient loads and delays.

Both negative and positive personal experiences shaped patients perspectives and attitudes, significantly influencing their access to healthcare services. The PwDM expressed mistrust in the healthcare system and the intent of the treating physicians, fearing unnecessary lifelong medications. ASHA explained that patients perceived healthcare initiatives as profit-driven, discouraging their participation. Mistrust in doctors, driven by perceptions of financial gain and reluctance to provide necessary treatments like anti-VEGF injections, spread negativity. This distrust discouraged timely eye care, leading patients to seek treatment only during emergencies.

#### Individual level barrier

The patients highlighted the emotional and physical challenges of living with severe vision loss. A patient shared her challenges of managing kitchen work while also dealing with loneliness and a lack of family support. Another described profound dependence on family members for basic tasks, such as using the washroom, due to difficulty seeing even short distances, emphasizing the loss of autonomy and added burden on caregivers. These challenges lead to emotional distress and reduced motivation, which deterred patients from seeking DRS.

#### Theme 3: cognitive barriers

Cognitive barriers refer to knowledge and communication challenges that emerge from the interactions and experiences of healthcare professionals, patients, and their families with the healthcare system. These barriers impact healthcare utilization individually and collectively, leading to delayed care, lack of treatment, and poor health outcomes^33^.

#### Perceptions about doctor-patient relationships and communications

Patients reported satisfaction with their interactions with HCPs, including MO, ophthalmologists, and retina specialists, across primary healthcare, district hospitals, and tertiary care settings. They highlighted positive interactions with doctors, noting their compassion and professionalism, and emphasized their trust in the doctors’ clinical expertise and the care they received. These doctors took the time to explain patients’ conditions and discuss the treatment plans. However, reports of mistreatment from the security staff while in the waiting lines were also revealed. Previous negative experiences with healthcare professionals include perceptions of being ignored, shouted at, or spoken to in a derogatory manner. This fear often results in patients avoiding care or prematurely leaving hospitals without receiving adequate treatment.

#### Limited disease-related communication

Some ophthalmologists expressed concern that patients receive limited information about DR from diabetes treatment physicians. This lack of information delays DR screening, as patients are unaware of the disease’s potential consequences. The HCPs believe that the national blindness control programs emphasis on cataract management has overshadowed efforts to raise awareness about DR. The retina specialist indicated that primary healthcare providers often lack knowledge about diabetes complications, leading to inadequate awareness among diabetic patients.

#### Awareness, attitudes, and priorities in diabetes and DR

In our study, PwDM believed white sugar primarily causes diabetes and preferred jaggery as an alternative. Despite the understanding of DM and its effects on vital organs in some PwDM, there is limited awareness about DR.

The HCPs emphasize that a lack of basic education significantly impacts diabetic patients understanding and awareness of DR. Some patients even attribute their low vision to past birth-related misdeeds. Women display lower eye care awareness due to limited educational access, domestic confinement, and reduced exposure to reliable health information sources. Patients’ reluctance to disclose their diabetes history reflects a lack of awareness about the importance of managing DM and its complications (DR).

At the same time, the PwDM interaction with peers also shaped their decision to access healthcare facilities. Due to the high cost of treatment and multiple treatment visits, the DR patients advise others to forgo further treatment and manage with one eye due to perceived inefficacy. Many doubt the importance of eye care, with common thoughts like *“My vision is unaffected” or “There’s no need for an eye examination.”* They visit eye care facilities only when experiencing vision problems. This skepticism drives them to seek alternative methods, such as herbal remedies, for managing their eye conditions. The HCPs reported that in rural areas, people commonly use antibacterial chloramphenicol capsules *“mungre ankhon me dalte hai*,*”* honey, and kajal as alternative treatments for various eye conditions, including low vision. The ophthalmologists expressed concern over patients misattribution of low vision only to cataracts and sought minimal solutions like eye drops or spectacles. Despite ophthalmologists’ attempts to encourage patients to start and complete treatments for improved vision, many prioritize obtaining a *“disability certificate”* for government benefits, highlighting a lack of awareness and reluctance to seek essential care.

#### Health seeking behaviour

Although specialized eye care services are unavailable at local healthcare institutions, PwDM often visits these nearby facilities for convenience. The ophthalmologist stated that individuals seeking cataract surgery often did not disclose their diabetic status, which was usually detected only through preoperative blood tests.

#### Theme 4: psychological barriers

Psychological barriers, including inaccurate diagnoses, the conduct of healthcare personnel, and previous adverse experiences in healthcare settings, negatively impact healthcare access. Apprehension, skepticism, and negativity often shape individuals’ perspectives, impacting individuals healthcare-seeking to seek DR care^23^.

#### Fear and anxiety

The HCPs observed that individuals with vision loss from DR often experience heightened anxiety. This complicates efforts to persuade patients to prioritize DR treatment, especially when managing other comorbidities that demand immediate attention. An ophthalmologist noted that individuals with DR often experience anger and frustration due to vision loss, which hampers their ability to read, work, perform household tasks, and move independently. Work-related stress and wage loss exacerbate the burden of meeting family financial responsibilities, creating additional challenges for individuals with low vision. The perception of multiple treatment visits, associated costs, and fear of treatment failure despite the best efforts were major barriers to DRS screening access.

#### Hopelessness

Hopelessness arises as the individual with low vision led one individual to consider extreme measures, including suicide, reflecting a deep sense of hopelessness. Another PwDM faces distress over the potential impact of vision impairment on his family. The HCPs observed that individuals with DR often experience a deep sense of hopelessness due to physical dependency due to low vision, further discouraging seeking care. Patients with severe NPDR and PDR often feel hopeless, believing their vision loss is irreversible. This discourages them from seeking treatment, reflecting the emotional toll of advanced vision impairment and its impact on care engagement.

## Discussion

This qualitative study objective has provided insight into the barriers to accessing DRS from the perspectives of PwDM and HCPs across four themes of the HCAB^20^. Overall, participants described various interrelated factors contributing to their decision to decline or delay attending DRS services (Fig. 2)

In our study, the majority of PwDMs did not have any health insurance schemes. The PwDM were unaware of the PMJAY coverage^5^ for DRS and treatment. Similarly, a study conducted at Bascom Palmer Eye Institute found an awareness gap in health insurance coverage for DR^34^. Engaging private providers under public health insurance schemes is recommended to streamline referral management^5^. However, as indicated in the other study, the availability of health insurance schemes does not guarantee adequate coverage unless its awareness is optimal^35^. Our study identified significant financial barriers, particularly for PwDM over 60 years old who rely on family support. The retina specialist and the ophthalmologist explained that PwDM chose “daily wages” over “eye health,” which is influenced by the complex interplay between employment status and access to DRS services. Financial dependency on family support was more prevalent when the patient was the primary breadwinner^36^. Piyasena et al. noted that the fear of taking leave from work and reduced earnings led to a prioritization of income generation over DR care^37^.

Structural components, such as the unavailability of DRS equipment, an insufficiently trained workforce, and long waiting times, precluded access to DRS in public health facilities in our study. The uneven distribution of skilled workforce (ophthalmologists and optometrists) at the primary and secondary levels of care leads to referral and overcrowding at tertiary healthcare centres. Evidence highlights the need to assess public health facilities for DRS, including equipment for diagnosis and treatment, staff availability, and skill levels^36,37^. The PwDM often questioned the need for multiple eye tests, hesitating to seek care due to concerns about prolonged hospital stays. Concerns about being prescribed “unnecessary” medication in the absence of symptoms were also highlighted in a study conducted in South India.

Reference^19^ The ophthalmologists and optometrists in our study recommended training non-ophthalmologists, such as nurses, optometrists, and other para-ophthalmic employees, adopting a “task-shifting” approach for DRS, enhancing accessibility, and optimizing specialists to focus on managing referred patients for treatment, a strategy well-documented in other studies^5,38–41^.

The study highlighted the lack of awareness about DR as a preventable cause of blindness. Its asymptomatic nature presents a key barrier, making it difficult for PwDM to grasp the urgency and importance of DRS. Likewise, limited awareness and failure to associate DM with its complications often lead to the need for DRS being overlooked until vision deteriorates^19,42,43^. The HCPs emphasized limited awareness of DRS among ASHAs, CHOs, and other community health workers, recommending education on DM and its complications while leveraging their community networks. Counseling initiatives, though, have been shown to improve awareness, enhance quality of life, and promote adherence to screening and treatment^44,45^. However, other studies have highlighted that counseling PwDM was a low priority for healthcare professionals^8,37,46^.

The lack of awareness about DR led to underestimating its severity. Local remedies like “Munger” (chloramphenicol capsules), *“kajal”* (eyeliners), or *“shehad”* (honey) are used to treat their low vision. Low vision was more commonly attributed to cataracts and refractive errors rather than DR, as reported in a similar study from Southern India^19^. Limited DR awareness, along with financial constraints and old age, led PwDM to prioritize obtaining a *“disability certificate”* for a government pension over screening and treatment. A study from Sri Lanka also reported delays in seeking care were influenced by patients’ circumstances, highlighting the severity of the situation, where systemic health conditions took precedence over eye care^37^.

Diabetic participants expressed concerns about taking lifelong prescribed medications and viewed anti-VEGF injections as harmful *“chemicals”.* Consequently, many sought care from unqualified practitioners or relied on local remedies of unknown composition, often leading to severe eye complications. DR diagnosis caused significant stress among some patients, with some even contemplating suicide.

Our study identified limited doctor-patient interaction and communication as a key factor affecting DRS access. PwDM cited rude and discriminatory staff behavior as reasons for discontinuing care, while ophthalmologists observed that many PwDM, especially those with lower literacy, struggle to grasp the importance of regular blood sugar monitoring and eye screenings. In another study, patient dissatisfaction was linked to the perception of inadequate explanations or doctors rushing due to busy schedules^23^. While men often serve as primary decision-makers for healthcare access for female family members^37^, our study found that older men and women primarily relied on family members for decision-making and reported limited access to DRS. Gender-specific disparities in DR care access were not explored further in interviews, suggesting this is an area for additional research.

Synthesizing the evidence pinpointed modifiable and non-modifiable barriers to identifying themes in DRS access. Knowledge gaps about DR among PwDM were identified as a modifiable barrier. At the same time, lack of health insurance, low income, and financial constraints were considered non-modifiable barriers at the health system level (although they may be more amenable to modification at the macro-economic level). Future research should focus on ensuring inclusivity and developing a robust framework for successful DRS integration into general practice. Addressing these issues in policy and practice, targeted interventions can enhance DRS attendance.

### Strength

Our study employed a theory-informed approach, using the HCAB model to pinpoint measurable and modifiable barriers to DRS, focusing on causal pathways and adverse health outcomes. This study effectively highlighted the patients’ and HCPs perspectives, highlighting the real-world challenges to DRS. Patient and provider insights provided valuable cues for developing effective intervention strategies. This is the first study conducted in North India that examines the barriers encountered in DRS in public health facilities, corroborating findings from similar studies nationwide^10,19,47^.

### Limitations

A larger sample size of PwDM from neighbouring North Indian states would have contributed to a more comprehensive understanding of the prevailing DRS situation across the entire region. Interviews with family members, essential in decision-making on care-seeking and supporting patients, would have been advantageous for the study.

## Conclusion

Managing diabetes and its complications, such as low vision, particularly in cases of VTDR, is a lifelong challenge. This study identified key themes at user and healthcare provider levels, highlighting areas for improvement in implementing DRS in public health settings. Addressing modifiable barriers, such as limited awareness among PwDM about diabetes and its complications, through health promotion strategies is crucial. Educating primary care doctors on informing patients about diabetes-related complications remains essential. Findings emphasize integrating DRS with awareness campaigns to enhance compliance and improve care outcomes. This study, along with others from India^41,48^, recommends tailored DRS services, including outreach programs, to effectively address patients specific circumstances. Health insurance awareness can motivate patients to access eye care services and improve follow-up compliance, enhancing vision outcomes.

## Electronic supplementary material

Below is the link to the electronic supplementary material.

Supplementary Material 1

## Data Availability

The datasets generated and/or analyzed during the current study are not publicly available due to ethical considerations and confidentiality agreements related to state health systems data but are available from the corresponding author at a reasonable request.
