# Supplementary material for: Exploring patient and health care provider perspectives on barriers to diabetic retinopathy screening in public health facilities in North India
Source: Sci Rep. 2025 Mar 10;15:8251. doi: 10.1038/s41598-025-92795-y (PMC11894220; doi:10.1038/s41598-025-92795-y)
Supplement: Supplementary file 1 — Supplementary Material 1 [file 41598_2025_92795_MOESM1_ESM.docx]

**Title: Exploring Patient and Health Care Provider Perspectives on Barriers to Diabetic Retinopathy Screening in Public Health Facilities in North India**


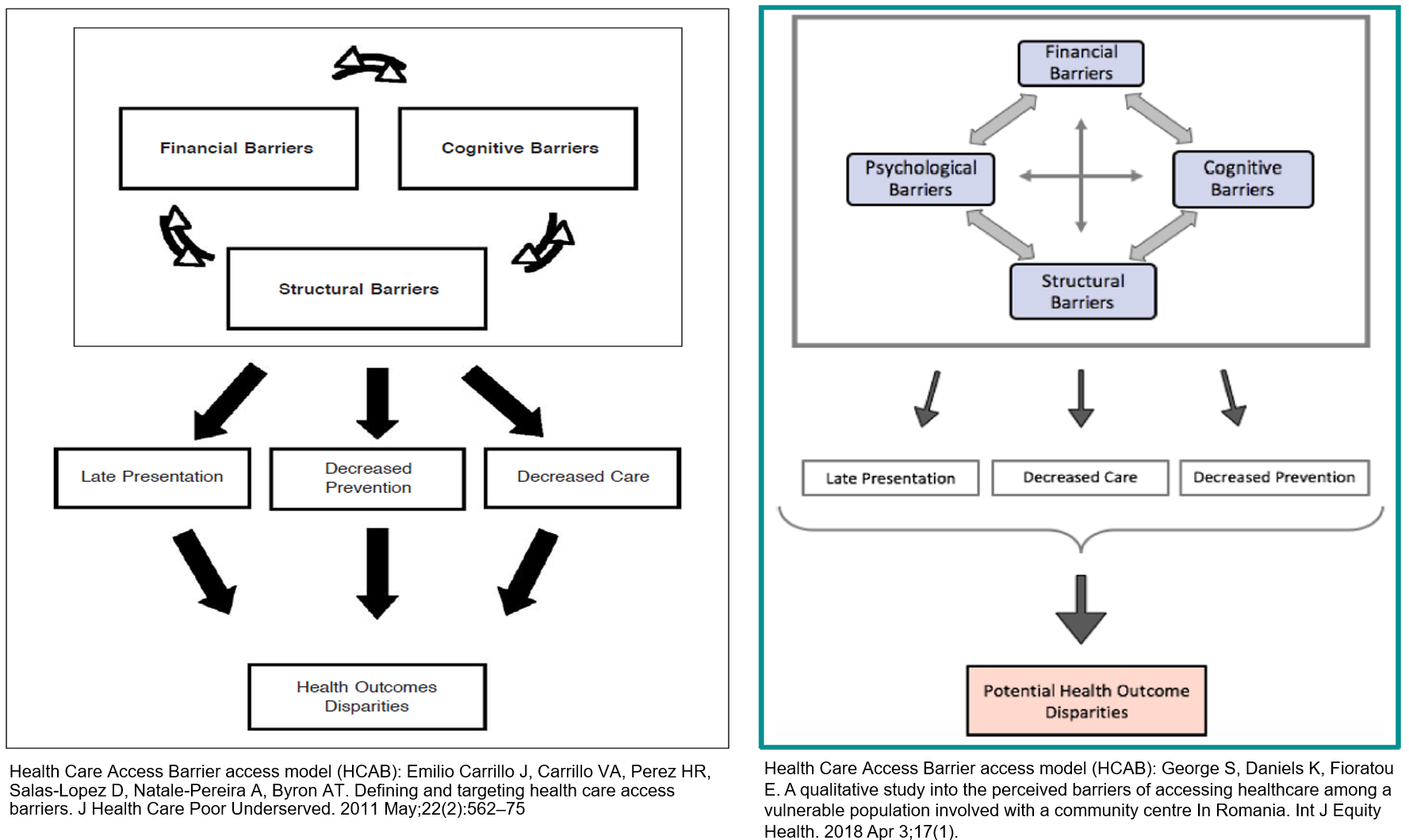


Supplementary Figure 1a and 1b: Original HCAB model displaying the four themes (financial, structural, cognitive, psychological) adapted in the present study

Supplementary Table 1: Consolidated criteria for reporting qualitative studies (COREQ): 32-item checklist

| **No. Item** | **Guide questions/description** | **Manuscript**  **(section and page)** |
| --- | --- | --- |
| **Domain 1: Research team and reﬂexivity** |  |  |
| *Personal Characteristics* |  |  |
| 1. Interviewer/facilitator | Which author/s conducted the in-depth interviews? | AC and MS conducted the interviews.  Data collection, p 5 |
| 2. Credentials | What were the researcher’s credentials? E.g. PhD, MD | Master of public health and one pursuing doctoral studies.  Data collection, p 5 |
| 3. Occupation | What was their occupation at the time of the study? | AC: Research associate  MS: Field investigator  Data collection, p 5 |
| 4. Gender | Was the researcher male or female? | AC and MS were both males.  Data collection, p 5 |
| 5. Experience and training | What experience or training did the researcher have? | AC and MS both have more than 5 years of conducting qualitative research experience.  Data collection, p 5 |
| ***Relationship with participants*** |  |  |
| 6. Relationship established | Was a relationship established prior to study commencement? | Obtaining permission and building rapport with the participants of the study were established. |
| 7. Participant knowledge of the interviewer | What did the participants know about the researcher? E.g., personal goals, reasons for doing the research | The researchers explained the study procedures and objectives before enrolment in the study. |
| 8. Interviewer characteristics | What characteristics were reported about the interviewer/facilitator? E.g., Bias, assumptions, reasons and interests in the research topic | The researchers interests in the research topic were explained to the participants. |
| **Domain 2: study design** |  |  |
| *Theoretical framework* |  |  |
| 9. Methodological orientation and Theory | What methodological orientation was stated to underpin the study? e.g. grounded theory, discourse analysis, ethnography, phenomenology, content analysis | Braun and Clarke's six-phase approach to performing thematic analysis for data anaysis  Data analysis, p 5 |
| *Participant selection* |  |  |
| 10. Sampling | How were participants selected? e.g. purposive, convenience, consecutive, snowball | Study participants and sampling.  p 4 |
| 11. Method of approach | How were participants approached? e.g. face-to-face, telephone, mail, email | Study participants and sampling.  p 4  Data collection  p 5 |
| 12. Sample size | How many participants were in the study? | Study participants and sampling.  p 4  Data collection  p 5 |
| 13. Non-participation | How many people refused to participate or dropped out? Reasons? | Study participants and sampling.  p 4  Data collection  p 5 |
| *Setting* |  |  |
| 14. Setting of data collection | Where was the data collected? e.g. home, clinic, workplace | Data collection  p 5 |
| 15. Presence of non-participants | Was anyone else present besides the participants and researchers? | No one was present except the participant and the interviewer.  Data collection  p 5 |
| 16. Description of sample | What are the important characteristics of the sample? e.g. demographic data, date | Participant characteristics  p 6 |
| *Data collection* |  |  |
| 17. Interview guide | Were questions, prompts, guides provided by the authors? Was it pilot tested? | Study design  p 4 |
| 18. Repeat interviews | Were repeat interviews carried out? If yes, how many? | Non-repeat interviews were conducted. |
| 19. Audio/visual recording | Did the research use audio or visual recording to collect the data? | Data collection,  P 5 |
| 20. Field notes | Were ﬁeld notes made during and/or after the interview? | During some interviews, notes were filed but not in a thorough manner. |
| 21. Duration | What was the duration of the in-depth interview? | Data collection,  p 5 |
| 22. Data saturation | Was data saturation discussed? | Recruitment was not based on data saturation. |
| 23. Transcripts returned | Were transcripts returned to participants for comment and/or correction? | No |
| **Domain 3: analysis and ﬁndings** |  |  |
| *Data analysis* |  |  |
| 24. Number of data coders | How many data coders coded the data? | Three coders (AC, SD, HR) coded the data. Experienced public health experts (MD, AK) merged codes with similar meanings to eliminate redundancy.  Data analysis,  p 5 |
| 25. Description of the coding tree | Did authors provide a description of the coding tree? | Not provided |
| 26. Derivation of themes | Were themes identiﬁed in advance or derived from the data? | Themes were identified through HCAB model that defines healthcare access.  Data analysis, p 5  Results, p 6-7 |
| 27. Software | What software, if applicable, was used to manage the data? | Atlas Ti 23 software was used to manage the data.  Data analysis, p 5 |
| 28. Participant checking | Did participants provide feedback on the ﬁndings? | Participants were not involved in the conduct of the study. |
| *Reporting* |  |  |
| 29. Quotations presented | Were participant quotations presented to illustrate the themes/ﬁndings? Was each quotation identiﬁed? e.g. participant number | Due to the manuscript word limit, quotes under HCAB themes and sub-themes are provided as  supplementary table 6. |
| 30. Data and ﬁndings consistent | Was there consistency between the data presented and the ﬁndings? | Yes the data was presented under already defined HCAB themes, |
| 31. Clarity of major themes | Were major themes clearly presented in the ﬁndings? | Yes, all the HCAB themes, financial, structural, cognitive, and psychological themes, are presented clearly in the results. |
| 32. Clarity of minor themes | Is there a description of diverse cases or discussion of minor themes? | Data analysis: deductive analysis for presentation of already existing HCAB themes and inductive analysis for new emergent themes.  Data analysis, p 5 |

Supplementary Table 2: Examples of the words modified after piloting

| Before pilot | English | Diabetes mellitus | Diabetic retinopathy | Health insurance | Blurring of vision |
| --- | --- | --- | --- | --- | --- |
|  | Hindi | मधुमेह | आँखों की बीमारी | हेल्थ बीमा /इन्शुरन्स | धुंदला दिखना |
|  | Punjabi | ਸ਼ੂਗਰ | ਅੱਖ ਦੀ ਬਿਮਾਰੀ | ਸਿਹਤ ਬੀਮਾ | ਧੁੰਦਲੀ ਨਜ਼ਰ ਦਾ |
| After pilot | English | Sugar | Sugar-related eye problem | Ayushman Bharat card | - |
|  | Hindi | शुगर या मीठे की बीमारी | शुगर सम्बंधित आँखों में दिक्कत | पांच लाख वाला कार्ड | निगाह कमजोर |
|  | Punjabi | ਨਜ਼ਰ ਦਾ ਧੁੰਦਲਾ ਹੋਣਾ | ਨਜ਼ਰ ਦਾ ਧੁੰਦਲਾ ਹੋਣਾ | ਨਜ਼ਰ ਦਾ ਧੁੰਦਲਾ ਹੋਣਾ | ਨਜ਼ਰ ਦਾ ਧੁੰਦਲਾ ਹੋਣਾ |

Supplementary Table 3: Inclusion criteria for study participants

| **Inclusion criteria for PwDM** | **Inclusion criteria for HCP** |
| --- | --- |
| Male or female, aged 30 years or older | Male or female, aged 30 years or older |
| Diagnosed DM irrespective of duration of diabetes | Those involved in DM and eye care diagnosis and treatment in public health systems (community, primary health centre, and tertiary healthcare centre) |
| Able to communicate in Hindi or Punjabi | Able to communicate in Hindi, Punjabi or English |
| Able to give consent to participate in the interview | Able to give consent to participate in the interview |

***HCP - Healthcare Provider, PwDM - People with Diabetes Mellitus

Supplementary Table 4: Characteristics of people with diabetes mellitus

| **Sociodemographic Characteristics of Patients** | **n (%)** |
| --- | --- |
| **Gender** | |
| Male | 15 (58%) |
| Female | 11 (42%) |
| **Age (years)** | |
| 30-40 | 3 (11.5) |
| 41-50 | 7 (30) |
| 51-60 | 7(27) |
| 61-70 | 7 30) |
| 71-80 | 2 (7.7) |
| **Marital status** | |
| Married | 24 (92) |
| Single  Widow/widower | 0(0)  2(8) |
| **Educational level** | |
| Non-literate | 12 (46) |
| Up to 5th standard | 6 (23) |
| Up to matriculation | 3 (11.5) |
| Up to 10+2 | 3 (11.5) |
| College and above | 2 (7.7) |
| **Occupation** | |
| Electrician | 2 (7.7) |
| Driver | 3 (11.5) |
| Self-employed | 5 (19.2) |
| Retired | 4 (15.4) |
| Not employed | 12 (46) |
| **Duration of Diabetes** | |
| Mean ± SD (yrs) | 11.9 ± 6.3 |

Supplementary Table 5: Characteristics of Healthcare providers

| **Characteristics of HCPs** | **n (%)** |
| --- | --- |
| **Gender**  Male  Female | 10 (52.6)  9 (47.4) |
| **Age (years)**  20-30  31-40  41-50 | 1 (5)  12 (63)  6 (31.5) |
| **Professional status**  Retina specialist  Ophthalmologist  Medical officer  Optometrist  CHO  ASHA | 2 (10.5)  3 (15.8)  2 (10.5)  5 (26.3)  3 (17.4)  4 (22) |

***ASHA - Accredited Social Health Activist, CHO - Community Health Officer; HCP - Health Care Provider

Supplementary Table 6: Themes and examples of participants' quotations (Patient and HCPs Perspective)

| **Theme** | **Sub-theme** | **Sample of illustrative quotes** | **Participants**  **PwDM (n = 26)**  **HCP (n = 19)** |
| --- | --- | --- | --- |
| **Financial Barriers** | Affordability  (Cost) | *I have little income and cannot afford treatment even at public hospitals. To even visit (name of the facility) costs Rs 30 one way via auto from my home, so the medicine cost is extra. The only option is to sit at home and wait for God's mercy.* ***P5***  *“Cost is the most significant barrier. Patient visits will be infrequent if they have a poor economic background.”* ***Optom 1***  *“In public hospitals, people think we will get medicine and glasses for free. They stop coming for the next appointment once you ask them to pay for something else.”* ***Optom 4*** | PwDM = 17  Opt = 3  Optom = 5  RS =1 |
|  | Affordability  (Employment status) | *The money problem is the biggest issue. I work as a driver, and I get Rs 300-400 per day; I can either come for an eye check or feed my family; I have to make choices.* ***P12***  *Most patients visiting my OPD are daily wagers* ***(roz kamake khane wale)*** *and only visit a few times, as they miss their one-day wage.* ***Opt2*** | PwDM = 14  Opt = 3  Optom = 2  RS =1 |
|  | Affordability  (Health Insurance) | *No, I do not have an Ayushman Bharat card* ***(panch lakh alla card)****; my neighbors said you get reimbursed only if admitted to the hospital.* ***P4***  *“Since they (private health insurance company) learned about my diabetes and poor vision, they denied saying I am ineligible for the health insurance.”* ***P3***  *DR screening services are covered under AB, but the treating clinician needs to be made aware of its coverage along with the patients.* ***Opt 3*** | PwDM = 2  Opt = 3 |
| **Structural Barriers** | Health system barriers  (Service availability & delivery, barriers, accessibility,  long waiting hours) | *Here (name of the facility), the doctors are skilled in treating retina (parda dekh sakte hai), which is not available in my area; the (name of the facility) needs more expertise, and I have to visit here.* ***P18***  *There is an accumulation of doctors in one place at the district hospital but not at the primary level.* ***Opt3***  *I cannot come so far for my treatment in a local bus that operates between scheduled times during the day. If I miss the bus, I do not have money to arrange my stay in the city.* ***P8***  *If a patient plans to visit (name of the facility), he has to leave his home at 4:00 a.m. and reach here at 8:00 a.m. for registration. That means 4 hours, and he must also return the same day.* ***RS2***  *The patient has to stand for a long time, and sometimes, it takes a whole day to meet the eye doctor, which is torture for old patients. I have a knee problem, which makes it unbearable to stand.* ***P12***  *Again, due to the huge rush, the patient will only get a treatment appointment on the same day if it is an emergency; he will get further dates for his tests. When we give the next appointment for diagnostic tests, they show frustration and say we will get blind in the next 2 or 3 days, and you are giving me a 2 to 3 months date.* ***RS2***  *What will I do there, "main uthe jaake ki karna" they will put me on unnecessary medicines for a lifetime.* ***P9***  *Some think we are doing this because we are minting money from these activities; what will we get if we go there "inha de ta apne pese bande aasi jaake ki karna”.* ***ASHA2***  *People say the doctor has put the money in their pocket, so they do not give injections (anti-VEGF). This spreads negatively, and people come for eye checks only if it is too urgent.* ***Opt1*** | PwDM = 10  Opt = 3  RS = 2  Optom = 5  MO = 2  ASHA = 4 |
|  | Individual barrier (Family support) | *I cannot see from one eye; I have to cook sometimes, and smoke goes into my eyes and creates problems. My son left me alone, and my daughter is married (starts crying).* ***P10***  *No, I keep sitting on the bed and doing nothing. I can barely see anyone, even at a 1-meter distance. I must remember about other work (Baareek kaam karna mushkil hai), so I must depend on my daughter-in-law to go to the washroom.* ***P3*** | PwDM = 10 |
| **Cognitive barriers** | Perceptions about doctor-patient relationships and communications | *"Even though the doctors are so busy, they take the time to explain the disease, its treatment, and what to expect. It really reassures us, and half of our worries just go away."* ***P4***  *“(Name of the hospital, tertiary hospital) is good for patients but not good for patient attendants. The non-medical/support staff, particularly the security staff, could be better, but actually, it is worse”.* ***P20*** | PwDM = 4 |
|  | Limited disease-related communication | *They told me not to eat white sugar, corn chapati, rice, and potato, but I* *was never informed about an eye check-up (menu kisi ne nahi daseya).* ***P5***  *Our weak point is that no one refers diabetic patients from peripheries for DR screening; even CHOs regularly see diabetic patients. But they never send any such patient to us, and please remember patients are never told to refer because they are unaware of it.* ***Optom 4***  *“The National Blindness Program has mainly focused on cataract raising awareness and providing treatment but diabetic retinopathy hasn’t been a priority."* **Opt2** | PwDM = 14  Opt = 3  Optom = 4  RS = 2 |
|  | Awareness and attitude about diabetes and DR | *I know diabetes occurs due to the intake of sugar. So I prefer jaggery rather than sugar in my tea.* ***P6***  *I used to feel something was wrong. I ignored and thought, "lagaya kala motiya utreya hoya ni te chasma." I might need spectacle correction or have cataracts.* ***P21***  *The individuals with low education link it to their past deeds," Ye pichle karmo ka fal hai, jo hoga vo dekha jaayega,****”*** *so this will happen.* ***CHO1***  *We must educate health workers first because they do not know what causes diabetic retinopathy, so they counsel the patient that only it will make a difference.* ***RS1***  *In the village, a common practice is using Chloramphenicol eye ointment or eyeliner/kajal as home remedies for eye-related issues instead of visiting a hospital for proper treatment.* **CHO1**  *During my visit to (name of the health facility), I interacted with some patients in the waiting room, and they told me we had received 5-10 injections.* *We wasted so much money, but the condition was still the same, and they suggested not getting any treatment and managing with one eye.* ***P16***  *They say, give us some eye drops or spectacles (wo itna he bolte hai thoda sa number de do jo hota hai”).* *We are happy with whatever vision we have****.*** ***Optom1***  *I try to convince them for treatment, “ilaaz ho jayga” then they say we are contented with whatever vision we have we only want our handicap certificate to get our government pension.* ***Opt1*** | PwDM = 14  Opt = 3  Optom = 5  RS = 2  MO = 2  CHO = 3 |
|  | Health seeking behaviour | *People hide their diabetic history. When we plan for cataract surgery and get the blood investigations done, only then do we find out that they have diabetes.* ***Opt3***  *People in villages want to keep their sugar history private.* ***ASHA4***  *Patients often choose nearby facilities and assume every facility is available under one roof. They later complained of the non-availability of services, irrespective of our instructions to visit the district hospital and tertiary eye care facility.* ***MO1*** | Opt = 3  MO = 2  CHO = 3  ASHA = 4 |
| **Psychological barriers** | Fear and anxiety | *One or two patients cry every day in my OPD after knowing that their vision will not be restored. Additionally, the co-morbidities coexist (nephropathy, neuropathy), so the anxiety level is too high.* ***RS1***  *I would not sleep for 24 hours and would sit over the phone. I was always angry (“Khaane ko padta tha”) and would abuse my family.* ***P3***  *I am always conscious and fearful while driving alone to the hospital. I used to go to the local market, but now I cannot go alone.* ***P13*** | PwDM = 4  RS = 1  Opt = 4  Optom = 1 |
|  | Hopelessness | *They informed me that my right eye had poor vision and another was almost 60% damaged. I got scared that I would be blind for the rest of my life, and I was thinking of committing suicide. I am a sufferer now, and my family has to suffer because of me****.*** ***P23***  *“Severe NPDR and PDR patients say nothing good will happen, so they surrender. They say we have reached an irreversible stage where we do not have any hopes to improve our vision”.* ***Optom 2*** | PwDM = 2  Opt = 2  Optom = 1  RS = 1 |

***ASHA - Accredited Social Health Activist, CHO - Community Health Officer, MO - Medical Officer, Opt - Ophthalmologist, Optom - Optometrist, OPD - Outpatient Department, P - Patient, PwDM - People with Diabetes Mellitus, RS - Retina Specialist
